# Office Visitors

**Published:** 1907-10

**Authors:** 


### OFFICE VISITORS.

We do not suppose any other professional men have as many and different kinds of visitors to their offices as do the dentists', we mean besides those who apply for professional services. We have lately had a variety that would furnish enough material for a modern dime novel writer. An errand boy whose father was in limbo for a boisterous drunk and whose mother and sisters were "out of bread" and he wanted us to lend him "just fifteen cents." He got it. Two Sisters of the Catholic Church in charge of a colored orphanage, very quietly and pathetically desired us to give any small sum for the care of these forsaken waifs in the swelling ranks of colored humanity. They got a quarter. A Salvation Army officer, in blue unifurm trimmed with red, pulled out a memorandum book on us and invited us to assist in carrying on the campaign against vice and to aid in maintaining the barracks. He got fifty cents. He was followed by a bright-eyed, quick-tongued, handsome female, who had just come from the great western earthquake and fire and wanted to sell three boxes of grease erasing mixture for fifty cents. She got the coin, of course. Did a Dentist ever refuse a bright-eyed woman anything?

Then the gold scrap man appeared and with his own scales weighed up our filings and this time we had a dollar or two to put into our pocket, we thought, but in reality they were taken out. We thought we were ahead one time, when a brother practitioner, who had compared weights and found the buyer had his O. K. to beat the seller, proved to us that we were mulcted to the amount of at least half a dollar. Then came the illustrated paper man with his elegant bound book of foreign views and atlas of art—the latter got us as we as a profession are artful. We discharged the contents of our pocket to the tune of several dollars and signed a contract to pay so much per month for a year. It was tiresome but we paid it. When the agent of the rival illustrated paper learned how easy we were he visited us and was politely shown to the threshold. In his wake was the lame soap peddler—two cakes for a quarter. We took two. Then the little imps of the Saturday Evening Post beseeched us—we nickled some and lemoned others. The only ones who threw anything our way were the Antiphlogistine man, who invited us to write a testimonial of it, the Sozodont samples distributor and the fellow who thought our liver, lites and kidneys were all out of kilter and gave a full supply of Hepatic Saline Salts to get them in accord and regular working order. We are training our office girl though to differentiate as to the visitors and she has caught on to them pretty, well especially picture and book agents, and has managed to dynamite the most of them and has saved our religion a good many times.

